# Immunotoxicological Evaluation of Corn Genetically Modified with *Bacillus thuringiensis Cry1Ah* Gene by a 30-Day Feeding Study in BALB/c Mice

**DOI:** 10.1371/journal.pone.0078566

**Published:** 2014-02-10

**Authors:** Yan Song, Chunlai Liang, Wei Wang, Jin Fang, Nana Sun, Xudong Jia, Ning Li

**Affiliations:** Key Laboratory of Food Safety Risk Assessment of Ministry of Health, China National Center for Food Safety Risk Assessment, Beijing, People's Republic of China; Cedars-Sinai Medical Center, United States of America

## Abstract

This study was to investigate the immunotoxicological potential of corn genetically modified (GM) with Bacillus thuringiensis (Bt) *Cry1Ah* gene in BALB/c mice. Female BALB/c mice were randomly assigned to one of the four groups: the negative control group, the parental corn group, the GM corn group and the positive control group with 10 mice per group. Mice in the GM corn group and the parental corn group were fed with diets containing 70% corresponding corn for 30 days. Mice in the negative control group and the positive control group were fed with AIN93G diet, administered with saline or 200 mg/kg of cyclophosphamide (CY) via intraperitoneal injection 24 h before the termination of the study, respectively. At the end of the study, the immunotoxicological effects of the GM corn were evaluated through immunopathology parameters including body and organ weights, hematology and clinical chemistry parameters, histological examination, peripheral blood lymphocytes phenotype; humoral immunity including antibody plaque-forming cell, serum immunoglobulin, cytokine and half hemolysis value; cellular immunity such as mitogen-induced splenocyte proliferation, cytotoxic T-lymphocyte reaction, delayed-type hypersensitivity reaction; non-specific immunity including phagocytic activities of phagocytes, natural killer cell activity. A single dose of cyclophosphamide (200 mg/kg bw) was found to have significant adverse effects on immunopathology, cellular immunity, and humoral immunity in mice. The corn genetically modified with *Bt Cry1Ah* gene is considered consistent with the parental corn in terms of immunopathology, humoral immunity, cellular immunity and non-specific immunity. No adverse immunotoxicological effects of GM corn with *Bt Cry1Ah* gene were found when feeding mice for 30 days.

## Introduction

Genetically modified plant has been one of the most rapidly adopted technologies in the history of agriculture [1]. Since the introduction of the first genetically modified plant in the 1983, genetic engineering techniques and their applications have developed rapidly. Commercial cultivation of GM crops started in 1996 with approximately 1.7 million hectares, then expanded to more than 160 million hectares in 2011 representing 47% of soybean, 32% of corn, 15% of cotton, 5% of canola cultivation [2].

Genetically modified crops are carryinging novel traits including insect resistance, disease resistance, quality improvement, herbicide resistance [3]–[5]. However, the safety of genetically modified foods is still the focus of the public attention. In addition to the common safety problems of general food, genetically modified crops and their products may also have their unique ones. Up to now, in the guidelines established by Codex Alimentarius Commission (CAC), Organisation for Economic Co-operation and Development (OECD), and other international organizations and countries, the primary focus for safety assessment of GM crops is on evaluating general toxicity and allergenicity of the introduced protein(s), whereas general immunotoxicological investigations of whole GMOs are not described.

In the past decades, some immunotoxicology studies on GM crops or the proteins expressed by exogenous genes were conducted worldwide. In the European Commission project SAFOTEST (New methods for the safety testing of transgenic food), immunotoxicological effects of transgenic rice were assessed [6]. Moreover there were a few adverse evidences of genetically modified crops on immune system. Finamore's study observed that maize genetically modified with Bacillus thuringiensis(Bt) *Cry1Ab* gene induced alterations in the percentage of T and B cells and of CD4^+^, CD8^+^, T subpopulations, as well as increases of serum IL-6, IL-13, IL-12p70, and MIP-1beta in mice fed for 30 or 90 days [7]. A health survey showed that exposure to Bt sprays may lead to the allergic skin sensitization and induction of IgE and IgG antibodies to Bt protein in farm workers who had sprayed pesticides [8]. These studies implied that general immunotoxicological examinations of genetically modified crop and Bt protein may be considered to some extent.

The aim of this study, embedded in China National GMO Project of New Varieties, was to investigate the potential immunotoxicological effects of GM crops. The corn modified with *Cry1Ah* gene was developed by Institute of Plant Protection, China Academy of Agricultural Sciences Research. The *Cry1Ah* gene was cloned from the insecticidal protein genes of Chinese Bacillus thuringiensis isolate BT8. A plant expression vector pHUAh harboring the *cry1Ah* gene was constructed and transferred into the parental corn as described in Yue et al [9]. Coded protein Bt is a well-known insecticide to lepidoptera, diptera, and many other kinds of insects [10]–[11]. Bt can bind with the receptor of brush border membrane vesicles of insect's mid-gut, then lead to intestinal perforation, cell disintegrating and produce insecticidal action [12]. The parental corn used in this study has a long history of consumption in China, and there was no evidence of toxicity effects. In the study, corn flours from the GM corn and the parental corn were formulated into balanced basic AIN93G diets [13] at a proportion of 70%, then fed to BALB/c mice.

## Materials and Methods

### 1. Materials

The corn genetically modified with Bt *Cry1Ah* gene was developed by Institute of Plant Protection, Chinese Academy of Agricultural Sciences (Beijing, China). The planted seeds derived from the GM corn (Zea mays Line T5) and its corresponding parental corn (Zea mays Line Syn31) were grown simultaneously in the same conditions without pesticides treated in the test fields in Chinese Academy of Agricultural Sciences (Beijing, China). The main components and anti-nutrients of the GM corn flour and the parental corn flour were detected by Institute of Plant Protection, Chinese Academy of Agricultural Sciences (Table 1). The AIN93G diet and the diets containing GM corn flours or parental corn flours were formulated and purchased from HFK bioscience Co., Ltd. (Beijing, China). Cyclophosphamide was purchased from Jiangsu Hengrui Medicine Co., Ltd.(Jiangsu, China). Sheep red blood cells (SRBC) were purchased from Beijing Laboratory Biology technology Co., Ltd. (Beijing, China). FITC hamster anti-mouse CD3e, APC rat anti-mouse CD19, PE rat anti-mouse CD49b, APC rat anti-mouse CD4, PE rat anti-mouse CD8a, lysing buffer and Mouse CBA flex sets(Il-2, Il-4, Il-5, Il-10,IFN-γ, TNF) were purchased from Becton, Dicknson and Company (Franklin Lakes, NJ, USA). Mouse IgG, IgA and IgM ELISA kits were purchased from GenWay Biotech,Inc. (San Diego, CA, USA). Concanavalin A (ConA) and Lipopolysaccharide (LPS) were purchased from Sigma-Aldrich Co. LLC (St. Louis, MO, USA). Thiazoyl blue tetrazolium bromide was from Amresco LLC (Solon, OH, USA). RPMI 1640 medium, Hanks' balanced salt solution (HBSS), fetal bovine serum, phosphate buffered saline (PBS) and penicillin-streptomycin solution were from Thermo Fisher Scientific Inc (Logan, UT, USA).

**Table 1:** Main nutrients and anti-nutrients in the parental corn flour and the GM corn flours.

Main nutrients and anti-nutrients		Parental corn flour	GM corn flour
Amino acids, g/100 g		9.03	9.35
Protein, g/100 g		9.30	10.0
Fat, g/100 g		3.1	3.4
Carbohydrate, g/100 g		61.7	58.2
Moisture, %		6.99	7.41
Ash, %		1.28	1.53
Total dietary fiber, g/100 g		17.6	19.5
Copper, mg/kg		1.33	1.65
Magnesium, mg/kg		1100	974
Manganese, mg/kg		5.26	4.32
Zinc, mg/kg		21.3	23.0
Potassium, g/kg		1.59	1.50
β-carotene, mg/kg		<5.0	<5.0
Vitamin A, mg/kg		<0.1	<0.1
Vitamin B1, mg/kg		0.34	0.55
Folic Acid, µg/100 g		16.5	18.7
Fatty acids	Palmitic acid C16:0, g/100 g	0.54	0.54
	Oleic acid C18:ln9c, g/100 g	1.12	1.12
	Linoleic acid C18:2n6c, g/100 g	2.35	2.30
	α-Linolenic acid C18:3n3, g/100 g	0.011	0.011
	γ-Linolenic acid C18:3n6, g/100 g	0.014	0.014
	Cis-11-Eicosenoic acids C20:1, g/100 g	0.036	0.027
	EPA C20:5n3, g/100 g	0.019	0.018
Raffinose, g/100 g		<0.4	<0.4
Phylic acid, g/100 g		1.24	1.28
trypsin inhibitor, TIU/mg		2.39	2.42

### 2. Animals

Female BALB/c mice, SPF grade, 6–8 weeks old with body weight of 18–22 g were used for the study, and guinea pigs (250 g) were used for the preparation of complement for plaque-forming cell assay and hemolysis test. The animals were purchased from Beijing HFK Bioscience Co., Ltd. (Beijing, China). The animals were maintained in a controlled environment at a temperature of 20–25°C, relative humidity of 40–70%, artificially illuminated with a 12 hour light/dark cycle and air exchanges of 10–15 times per hour. All animals were examined for clinical signs of ill health on receipt and observed within 7 days of arrival. The study was approved by Animal Experimental Welfare & Ethical Inspection Committee, National Institute for Nutrition and Food Safety, Chinese Center for Disease Control and Prevention. Animal experiments and housing procedures were carried out in accordance with the laboratory animal administration rules of the Ministry of Science and Technology of the People's Republic of China.

### 3. Study design

Mice were randomly assigned to one of the four groups (the GM corn group group, the parental corn group, the negative control and the positive control group) with 10 mice per group based on body weight. Mice in the GM corn group and the parental corn group were fed with a diet containing of 70% corresponding corn, which was designed to achieve the highest concentration of Bt protein in the GM corn group as much as possible. Another two groups of mice fed with AIN93G diet were set as the negative control and the positive control (intraperitoneal injection with saline or 200 mg/kg bw of CY by 24 h before the termination of the study, respectively) (as shown in Table 2). Diets were adjusted to confirm the same amount of macronutrients in all groups and thereby avoid effects caused by compositional differences among the diets. The main nutrient compositions of diets were analyzed by Pony Testing International Group (Beijing, China) (Table 3). Mice were housed by group with ad libitum access to water and corresponding diets for 30 days.

**Table 2:** The design of experimental groups.

Group	Diet	Intraperitoneal Injection on Day 29
A	AIN93G standard	Saline
B	70% Parental corn	none
C	70% GM corn	none
D	AIN93G standard	cyclophosphamide

**Table 3:** Main nutrients and pollutants in the diets.

Main nutrients and pollutants	Negative/positive control	Parental corn	GM corn
Crude protein(%)	19.42	20.81	20.95
Crude fat(%)	10.07	7.88	7.53
Crude ash(%)	6.30	6.19	6.62
Total carbohydrate(%)	7.15	7.11	6.78
Crude fibre(%)	2.39	1.30	1.51
Calcium(%)	1.36	1.22	1.25
Phosphorus(%)	0.80	0.84	0.94
Lead(mg/kg)	0.21	0.35	0.35
Arsenic(mg/kg)	0.26	/	0.09
Mercury(mg/kg)	0.018	0.02	/
Cadimium(mg/kg)	0.13	0.19	0.19
aflatoxin(µg/kg)	9.42	10.82	12.40
benzene hexachloride(mg/kg)	/	/	/
DDT(mg/kg)	/	/	/

(note:“/” : <0.01 mg/kg).

Several 30-day feeding substudies were conducted in order to evaluate different immunotoxic effects. At termination, all animals were anaesthetized by carbon dioxide inhalation and killed by exsanguinations. The parameters of immunopathology, humoral immunity, cellular immunity, non-specific immunity were measured in the four groups with 10 mice per group. The study was approved by the Animal Ethics Committee of National Institute for Nutrition and Food Safety, Chinese Center for Disease Control and Prevention.

### 4. Preparation of spleen cell suspensions

The spleens were aseptically removed and transferred to culture dishes containing 2 ml of cold HBSS. Forceps and carbasus were used to finely triturate the spleens, then the cell suspensions were transferred to tubes and washed twice in HBSS and centrifuged for 10 min at 1000 rpm at 4°C. Splenocytes were resuspended in 3 ml of appropriate culture medium (Roswell Park Memorial Institute, RPMI, 1640 medium with phenol red supplemented) (Gibco, Grand Island, NY, USA). Cell numbers were determined for each splenocyte suspension by counting in a hemocytometer and cell viabilities were verified by trypan blue exclusion method.

### 5. Immunopathology

#### 5.1 Body weight and food intake

Each animal was observed twice daily for abnormalities, physical appearance and mortality. Body weight of mice was measured immediately prior to dosing, weekly thereafter, and at the end of the experiment. Food intake for each animal was determined weekly.

#### 5.2 Hematology and clinical chemistry parameters

For hematological analysis purpose, whole blood was collected from the retro-orbital plexus of each mouse in the presence of ethylenediaminetetraacetic acid (EDTA) anticoagulant. A COULTER Ac.T diff2 Hematology Analyzer (Beckman Coulter Corporation) was employed to measure the following parameters: total leukocyte count, differential leukocyte count and percentage, red blood cell count, hemoglobin, hematocrit, mean corpuscular volume (MCV), mean corpuscular hemoglobin concentration (MCHC), mean corpuscular hemoglobin (MCH), platelet count, red blood cell distribution width (RDW), mean platelet volume (MPV), prothrombin consumption test (PCT).

Blood for clinical chemistry analysis was collected into tubes without anticoagulant and centrifuged to obtain serum. Serum chemistry parameters included: alanine aminotransferase (ALT), aspartate aminotransferase (AST), total protein (TP), albumin (ALB), ratio of albumin to globulin (A/G), glucose (GLU), urea nitrogen (BUN), creatinine (CRE), cholesterol (CHO), triglyceride (TG), alkaline phosphatase (ALP), calcium (CA), sodium (NA), potassium (K). Parameters were analyzed using an automatic clinical analyzer (Hitachi 7080, Hitachi High-Technologies Corporation).

#### 5.3. Peripheral blood lymphocytes phenotyping

Whole blood was collected from the retro-orbital plexus of each animal in the presence of anticoagulant. Fifty µl of blood cell suspensions were stained with three-color combinations of antibodies of FITC hamster anti-mouse CD3e, APC rat anti-mouse CD19 and PE rat anti-mouse CD49b or antibodies of FITC hamster anti-mouse CD3e, APC rat anti-mouse CD4 and PE rat anti-mouse CD8 for 20 min at room temperature in the dark, then the blood samples were lysed with 2 ml of ammonium chloride-based lysing buffer for 20 min at room temperature in the dark and washed with 2 ml of PBS. Subsequently, the samples were resuspended in 0.5 ml of PBS and analyzed on FACSCalibur flow cytometer using CellQuest software (Becton, Dicknson and Company, USA). Appropriate isotype controls were used for compensation controls.

#### 5.4. Pathology

All mice were humanely sacrificed at termination, and a complete necropsy was performed. The following organs were removed and weighted: liver, kidney, spleen, thymus and lymph glands including cervical lymph nodes, axillary lymph nodes, mesenteric lymph nodes. Organ-to-body weight ratios (relative weight) were also calculated. The numbers of Peyer's patches on small intestine were counted. Macroscopic lesions were examined for each animal in all groups. All stored organs and tissues from all animals were embedded in paraffin, sectioned, stained with hematoxylin and eosin, and subjected to microscopic examination by the double-blind method.

### 6. Humoral immunity

#### 6.1. Plaque-forming cell (PFC) assay

The Jerne and Nordin antibody plaque-forming cell assay modified by Cunningham was performed [14]. Mice were immunized on day 25 with 0.2 ml of 2% (v/v) SRBC suspension in sterile saline via intraperitoneal injection. On day 30, the mice were sacrificed and spleen cell suspensions were prepared as stated in the part of Preparation of spleen cell suspension. Twenty-five µl of spleen cell suspension in RPMI 1640 medium supplemented with 10% fetal bovine serum and 1% penicillin-streptomycin solution (5×10^6^ cells/ml), 50 µl of 10% (v/v) SRBC in SA buffer solution (0.46 g of C_4_H_4_N_2_O_3_, 0.1 g of MgCl_2_·6H_2_O, 0.2 g of CaCl_2_·2H_2_O, 8.38 g of NaCl, 0.252 g of NaHCO_3_ and 0.3 g of C_8_H_11_N_2_NaO_3_ solved in 1000 ml stilled water) and 500 ul of agar solution (0.5 g/ml in HBSS, pH 7.2–7.4) were mixed in a glass tube, then poured onto slides. The slides were inverted on a special frame after the mixtures were solidified, and incubated at 37°C for 1.5 h, then guinea pig complement was added to the slot between the slides and the bottom of the frame. The slides were incubated at 37°C for another 1.5 h, then plaque production was enumerated and the results were expressed as the number of PFC per 10^6^ splenocytes.

#### 6.2. Serum immunoglobulin quantification

The animals were immunized with SRBC as stated in the PFC assay section. Total IgG, IgM, and IgA titers in the serum of each animal were determined by ELISA kits. The assay procedures were in accordance with the descriptions in the manufacturer's instructions. Briefly, 100 µl of standard, blank or diluted serum samples were added into the wells of 96-well plates in duplicate, the microtiter plates were covered and incubated at room temperature for 1 h, then washed 4 times with wash solution. One-hundred µl of enzyme-antibody conjugate was added into each well, and the plates were covered and incubated at room temperature for another 30 min, then washed 4 times with wash solution. Subsequently, 100 µl of TMB substrate solution was added into each well, and incubated at room temperature for 10 min. Then 100 µl of stop solution was added into each well. Finally the absorbance (450 nm) of the contents of each well was determined using an ELISA Reader (BioTek., USA).

#### 6.3. Hemolysis test

Mice were immunized with SRBC as stated in the PFC assay section. The sera were obtained and assayed for HC_50_. One ml of SA buffer solution, 0.5 ml of 10% (v/v) SRBC, 1 ml of diluted guinea pig complement (1∶8 diluted with SA buffer solution) and 2 ul of mouse serum were added into sample tubes. All tubes were kept in water bath for 15–30 min at 37°C, then the tubes were kept in ice bath to terminate the reaction. After centrifugated for 10 min at 2000 rpm, 1 ml of supernatant was collected and added into 3 ml of drabkin solution (1.0 g of NaHCO_3_, 0.05 g of KCN and 0.2 g of K_3_Fe(CN)_6_ solved in 1000 ml stilled water), at the same time, 0.25 ml of 10% (v/v) SRBC and 3.75 ml of Drabkin solution were mixed and placed for 10 min as positive control. The absorbance at 540 nm was detected, the HC_50_ value was got by the equation: HC_50_ = (OD_1_/OD_2_)×500 (OD_1_ = the OD value of sample well subtract that of control well, OD_2_ = the OD value of positive control well subtract that of control well).

#### 6.4. Serum cytokine levels

Whole blood was collected from retro-orbital plexus of each mouse without anticoagulant. Following centrifugation, the sera were obtained and assayed for the levels of cytokines including IL-2, IL-4, IL-5, IL-10, IFN-γ and TNF by Mouse CBA Flex sets. The assay procedure was in accordance with the descriptions in the manufacture's instruction. Briefly, 50 µl of mixed capture beads were added to each assay tube, and 50 µl of standard, blank or serum samples were added to the appropriate tubes, then PE detection reagent were added to each tube and incubated for 2 h at room temperature in the dark. The samples were washed with 1 ml of wash buffer, then resuspended in 300 µl of wash buffer and analyzed on FACSCalibur flow cytometer using CellQuest software.

### 7. Cellular immunity

#### 7.1. Mitogen-induced splenocyte proliferation

The mitogens used in this assay were ConA (100 µg/ml) and LPS (400 µg/ml), dissolved in distilled water. On day 30, the mice were sacrificed and the spleen cell suspensions were prepared as stated in part of Preparation of spleen cell suspension. One ml of spleen cell suspension (3×10^6^ cells/ml) was added into 24-well plate and cultured with 75 µl of the mitogen solutions or distilled water for control cultures. The plates were incubated at 37°C with 5% CO_2_ for 68 h, then 0.7 ml of supernatant in each well was discarded and 0.7 ml of RPMI 1640 medium was added, in the meanwhile, 50 µl of fresh prepared thiazoyl blue tetrazolium bromide solution (5 mg/ml, dissolved in PBS, pH 7.2) was added into each well, the plates were then incubated under the same conditions for an additional 4 h. Finally, 1 ml of acid-isopropanol (4 ml of 1 mol/L HCl added to 96 ml of isopropanol) was added into each well, the contents in each well were mixed thoroughly to make the purple crystallize fully dissolved and transferred to 96-well plates in triplicate, the absorbance at 570 nm was determined, and the results were calculated by subtracting the OD value of control well from OD value of sample well.

#### 7.2. Delayed-type hypersensitivity (DTH)

Mice were sensitized on day 25 via intraperitoneal injection of 0.2 ml of 2% (v/v) SRBC in sterile saline. Five days later, the thickness of left rear footpad of each mouse was determined with a vernier caliper (Changchun, Jilin Province, China). Then, the left rear footpad was injected with 20 µl of 20% (v/v) SRBC by subcutaneous injection. After 24 h, the thickness of left rear footpad was measured again. Swelling was expressed by the difference between two measurements before and after injection of SRBC to left rear footpad.

#### 7.3. Cytotoxic T-lymphocyte (CTL) assay

CTL assay was performed by lactate dehydrogenase-release assay [15]. On day 30, the mice were sacrificed and spleen cell suspensions were prepared as stated in part of Preparation of spleen cell suspension. Five-hundred µl of spleen cell suspension (effector cells, 6×10^7^ cells/ml) and 500 µl of P815 cell suspension (target cells, 1.2×10^6^ cells/ml) were added into 24-well round-bottom plate and cultured. Twenty-four hour cultures were collected, and the cell suspensions were transferred to tubes and washed in Hank's solution and centrifuged for 10 min at 200 rpm. The cells were resuspended in RPMI 1640 medium (6×10^7^ cells/ml). One-hundred µl of spleen cell suspension (effector cells, 2×10^7^ cells/ml) and 100 µl of P815 cell suspension (target cells, 2×10^5^ cells/ml) were added into 96-well round-bottom plate and cultured. Spontaneous LDH release and total LDH release of target cells as well as effector cell control were determined by addition of culture solution, 2.5%Triton solution and effector cell suspension, respectively. All tests were performed in triplicate. The plates were incubated at 37°C with 5% CO_2_ for 6 h. The plates were centrifuged at 1000 r/min for 5 min, then 100 µl of supernatant in each well was added into another 96-well flat-bottom plate, in the meanwhile, 50 µl of LDH freshly prepared LDH substrate solution was added to each well. The plates were incubated light-protected at room temperature for 30 min, and the reaction was stopped by adding 50 µl of 1 mol/L HCl/well. The absorbance at 492 nm was determined, and the results were calculated by the formula: % CTL cell activity = ((E−S−C)/(M−S))×100 (where E = experimental release of effector/target co-culture; S = spontaneous target cell LDH release; C = release of effector cell control; M = total target cell LDH release).

### 8. Non-specific immunity

#### 8.1. Carbon-clearance test in mice

The phagocytic activity of macrophage was performed as carbon-clearance test in mice [16]–[17]. Mice were injected with diluted ink (10 ml/kgbw) via caudal vein on day 30, at 2 min and 10 min after the injection, 20 µl of blood was collected from the retro-orbital plexus of each mouse and added into 2 ml of Na_2_CO_3_ solution (1 mg/ml), then the absorbance of the mixtures at 600 nm was measured. Index of phagocytosis was calculated by the equation: Index of phagocytosis = [body weight/(liver weight+spleen weight)]×[(lgOD_1_−lgOD_2_)/(t_2_−t_1_)]1/3 (OD_1_: the absorbance at 600 nm of 2 min after injection of ink, OD_2_: the absorbance at 600 nm of 10 min after injection of ink).

#### 8.2. Natural killer cell activity test

Natural killer cell activity test was performed by lactate dehydrogenase-release assay [18]–[19]. One-hundred µl of spleen cell suspension (effector cells, 2×10^7^ cells/ml) and 100 µl of YAC-1 cell suspension (target cells, 4×10^5^ cells/ml) were added into 96-well round-bottom plate and cultured. Spontaneous LDH release and total LDH release of target cells were determined by adding culture solution and 2.5%Triton solution, respectively. All tests were performed in triplicate. The plates were incubated at 37°C with 5% CO_2_ for 4 h. The plates were centrifuged at 1500 r/min for 5 min, then 100 µl of supernatant in each well was added into another 96-well flat-bottom plate, in the meanwhile, 100 µl of LDH freshly prepared LDH substrate solution (5×10^−2^ mol/L lithium lactate, 6.6×10^−4^ mol/L 2p-iodophenyl-3p-nitrophenyl tetrazolium chloride, 2.8×10^−4^ mol/L phenazine metosulphate, and 1.3×10^−3^ mol/L nicotineamide nucleotide NAD in 0.2 mol/L Tris-HCl buffer, pH 8.2) was added to each well. The plates were incubated light-protected at room temperature for 10 min, and the reaction was stopped by 30 µl of 1 mol/L HCl/well. The absorbance at 490 nm was determined, and the results were calculated by the formula: % NK cell activity = ((E−S)/(M−S))×100 (where E = experimental release of effector/target co-culture; S = spontaneous target cell LDH release; and M = total target cell LDH release).

### 9. Statistical analysis

All Statistical analyses were carried out using SPSS 17.0. The results were presented as means ± SD. Comparisons between multiple groups were carried out using one-way ANOVA followed by Bonferroni post hoc comparisons tests when equal variances was assumed and Dunnett's T3 post hoc tests when equal variances assumption was not met. Statistics with *p*-values less than 0.05 were considered significant.

## Results

### 1. Immunopathology

#### 1.1 Body weights, organ weights and food intake

**Figure 1:**
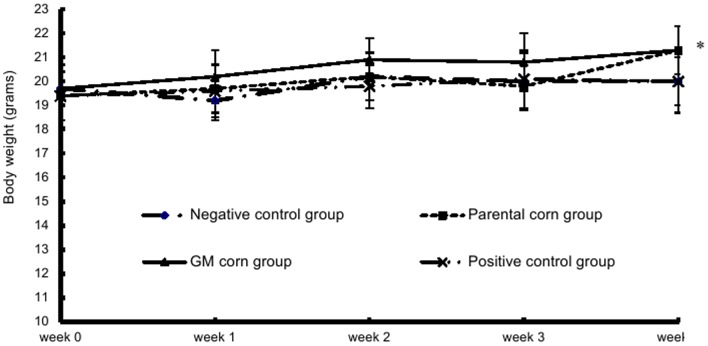
Mean weekly body weight of mice fed with diets containing GM corn and parental corn. Note: _*_: statistically significant different from the negative control group and the positive control group at p<0.05. The body weight of mice in the parental corn group and the GM corn group at week 4 were significantly higher than those in the negative control group and the positive control group.

**Figure 2:**
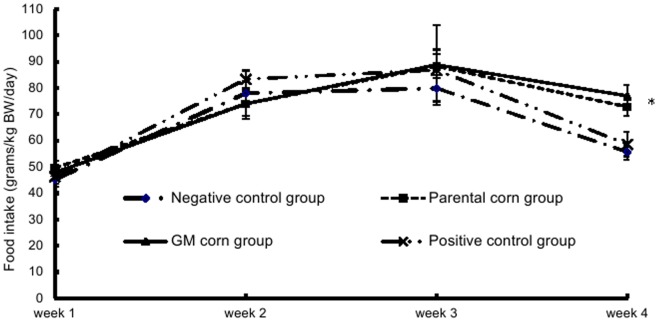
Mean weekly food intake of mice fed with diets containing GM corn and parental corn. Note: _*_: statistically significant different from the negative control group and the positive control group at p<0.05. The food intake of mice in the parental corn group and the GM corn group at week 4 were significantly greater than those in the negative control group and the positive control group.

The body weight and the food intake of mice in the parental corn group and the GM corn group at week 4 were significantly greater than those in the negative control group and the positive control group. No statistically significant differences of body weight and food intake were found among all groups in other weeks (Figure 1 and Figure 2).

As seen in Table 4, there was no significant difference in the number of Peyer's patches on small intestine among all groups. A single dose of CY (200 mg/kg bw) induced a significant reduction in absolute and relative weights of spleen, thymus and lymph glands of mice compared with those in the negative control group. No other significant differences were found when the comparisons were made among the GM corn group, the parental corn group and the negative control group.

**Table 4:** Effects on the number of Peyer's lymph gland and organ weights in mice after 30 days of feeding.

	Negative control	Parental corn	GM corn	Positive control
Number of Peyer's lymph gland	9.0±2.2	7.8±2.5	9.1±1.9	7.9±2.0
Liver				
Absolute weight (g)	0.719±0.064	0.723±0.155	0.731±0.116	0.708±0.427
Relative weight (%)	3.886±0.262	3.854±0.875	3.839±0.596	4.036±0.215
Kidney				
Absolute weight (g)	0.222±0.014	0.229±0.017	0.227±0.015	0.216±0.015
Relative weight (%)	1.201±0.076	1.213±0.075	1.194±0.082	1.228±0.074
Spleen				
Absolute weight (g)	0.071±0.018^d^	0.066±0.008^d^	0.066±0.007^d^	0.034±0.006^a^^,^ b ^,^ c
Relative weight (%)	0.382±0.084^d^	0.348±0.043^d^	0.344±0.020^d^	0.191±0.031^a^^,^ b ^,^ c
Thymus				
Absolute weight (g)	0.034±0.010^d^	0.036±0.007^d^	0.037±0.007^d^	0.021±0.007^a^^,^ b ^,^ c
Relative weight (%)	0.182±0.057^d^	0.193±0.040^d^	0.192±0.032^d^	0.118±0.041^a^^,^ b ^,^ c
Lymph gland				
Absolute weight (g)	0.009±0.005^d^	0.008±0.003	0.010±0.005^d^	0.005±0.003^a^^,^ c
Relative weight (%)	0.049±0.023^d^	0.041±0.015	0.052±0.027^d^	0.030±0.016^a^^,^ c

Note: Data are given as mean ± SD (n = 10).

a : statistically significant different from the negative control group at p<0.05;

b : statistically significant different from the parental corn group at p<0.05;

c : statistically significant different from the GM corn group at p<0.05;

d : statistically significant different from the positive control group at p<0.05.

#### 1.2. Hematology and clinical chemistry parameters

The mean WBC counts and differential leukocyte counts of LYM and MON, as well as the percentages of LYM and NEUT of mice in the positive control group were significantly lower than those in the negative control group, but the level of MPV in the positive control group was significantly higher than that in the negative control group. The counts of MON of mice in the GM corn group were significantly lower than that in the negative control group, whereas no statistical differences were seen when compared with the parental corn group (Table 5).

**Table 5:** Effects on haematology parameters in mice after 30 days of feeding.

Group	Negative control	Parental corn	GM corn	Positive control
RBC(×10^12^/L)	9.7±1.2	9.8±1.4	9.9±1.3	10.7±2.1
HGB(g/L)	155.9±18.5	162.2±22.4	160.0±19.4	176.3±37.3
HCT(%)	45.9±5.5	46.8±6.0	47.2±5.9	50.7±9.9
MCV(fL)	47.5±0.7	48.0±0.9	47.7±0.8	47.5±0.9
MCH(pg)	16.1±0.3	16.7±0.4	16.2±0.4	16.4±0.4
MCHC(g/L)	339.8±7.8	346.6±10.0	339.1±6.8	346.3±10.8
RDW(%)	13.3±0.3	13.8±0.3	13.5±0.4	13.6±0.4
PLT(×10^9^/L)	713.8±89.0	710.9±88.2	703.4±135.2	687.4±105.8
PCT(%)	0.18±0.03	0.19±0.05	0.19±0.04	0.20±0.05
MPV(fL)	2.5±0.4^d^	2.7±0.3	2.8±0.3	2.9±0.4^a^
PDW(%)	17.8±0.7	17.7±0.5	18.1±0.6	17.7±0.8
WBC(×10^9^/L)	7.4±1.8^d^	7.4±1.6^d^	7.5±2.1^d^	3.8±2.0^a^^,^ b ^,^ c
LYM(×10^9^/L)	5.4±1.2^d^	5.0±1.3^d^	5.4±1.6^d^	2.4±1.8^a^^,^ b ^,^ c
(%)	72.8±8.2^d^	66.0±4.9	70.6±3.6^d^	58.0±18.2^a^^,^ c
MON(×10^9^/L)	0.3±0.2^b^^,^ c ^,^ d	0.1±0.1^a^	0.1±0.2^a^	0.1±0.1^a^
(%)	4.4±4.5^b^	1.2±0.9^a^	1.5±1.6	3.0±4.5
NEUT(×10^9^/L)	1.7±1.1	2.4±0.4^d^	2.0±0.5	1.3±0.6^b^
(%)	22.1±8.6^d^	32.1±4.1	27.4±3.9	38.3±19.2^a^
EOS(×10^9^/L)	0.01±0.03	0.01±0.03	0.01±0.03	0.00±0.00
(%)	0.4±0.4	0.4±0.2	0.3±0.2	0.4±0.2
BAS(×10^9^/L)	0.00±0.00	0.01±0.03	0.00±0.00	0.00±0.00
(%)	0.4±0.2	0.3±0.3	0.1±0.2	0.3±0.4

Note: Data are given as mean ± SD (n = 10).

a : statistically significant different from the negative control group at p<0.05;

b : statistically significant different from the parental corn group at p<0.05;

c : statistically significant different from the GM corn group at p<0.05;

d : statistically significant different from the positive control group at p<0.05.

ALTs of Mice in the positive control group were significantly higher than those in the negative group. No other significant differences in clinical chemistry parameters were observed among all groups (Table 6).

**Table 6:** Effects on clinical chemistry parameters in mice after 30 days of feeding.

Group	Negative control	Parental corn	GM corn	Positive control
ALT(U/L)	27.0±3.9^d^	27.3±5.5	28.9±3.6	33.7±3.5^a^
AST(U/L)	148.6±29.0	141.6±31.6	165.9±20.2	165.4±31.2
TP(g/L)	65.66±5.27	66.37±3.94	64.72±5.82	65.62±3.49
ALB(g/L)	39.11±1.75	39.53±2.09	40.99±2.74	38.33±1.67
ALP(U/L)	113.7±12.6	125.1±18.7	121.9±14.5	114.6±10.8
A/G	1.51±0.28	1.41±0.12^b^	1.56±0.23	1.75±0.21^d^
GLU(mmol/L)	2.49±1.13	2.87±1.54	3.19±0.92	2.60±1.03
BUN(mmol/L)	8.64±1.32	8.33±1.07	8.20±1.51	8.78±1.13
CRE(µmol/L)	52.50±12.79	52.07±14.27	50.48±16.61	56.47±15.69
CHO(mmol/L)	2.06±0.27	2.26±0.22	2.11±0.20	2.20±0.24
TG(mmol/L)	1.10±0.24	1.12±0.22	1.04±0.21	1.14±0.20
Na(mmol/L)	131.1±6.9	132.7±15.7	141.0±16.3	135.4±16.5
K(mmol/L)	7.3±1.5	6.6±0.3	6.9±0.7	6.7±0.3
CA(mmol/L)	1.4±0.1	1.4±0.2	1.4±0.2	1.4±0.1

Note: Data are given as mean ± SD (n = 10).

a : statistically significant different from the negative control group at p<0.05;

b : statistically significant different from the parental corn group at p<0.05;

d : statistically significant different from the positive control group at p<0.05.

#### 1.3. Phenotypic analysis of peripheral blood lymphocytes

A single dose of CY (200 mg/kg bw) induced significant increases in percentage of B lymphocytes (CD3^−^CD19^+^), counts of CD4^+^ T lymphocytes(CD3^+^CD4^+^, Th cells) and ratio of CD4^+^/CD8^+^(Th/Ts) and a significant reduction in percentage of T lymphocytes (CD3^+^CD19^−^) when compared with those in the negative control group. There were no significant changes in percentages of T lymphocytes, B lymphocytes, NK cells (CD3^−^CD49^+^), Th cells, CD8^+^ T lymphocytes (CD3^+^CD8^+^, Ts cells) or Th/Ts ratio in the GM corn group when compared with the parental corn group and the negative control group (Table 7).

**Table 7:** Effects on phenotypic analysis of peripheral blood lymphocytes in mice after 30 days of feeding.

Group	CD_3_^−^CD_19_^+^ (%)	CD_3_^+^CD_19_^−^ (%)	CD_3_^−^CD_49_^+^ (%)	CD_3_^+^CD_4_^+^ (%)	CD_3_^+^CD_8_^+^ (%)	CD_4_^+^/CD_8_^+^
Negative control	59.5±8.6^d^	35.1±8.7^b^^,^ d	4.1±1.0	49.7±8.6^d^	9.8±1.0	5.1±1.0^d^
Parental corn	68.7±4.3^d^	24.8±4.9^a^^,^ d	5.4±0.7	57.8±4.5^d^	10.9±0.7	5.3±0.6^d^
GM corn	62.8±9.8^d^	28.2±10.9^d^	7.4±3.6	52.4±9.1^d^	10.4±1.7	5.1±1.0^d^
Positive control	84.6±7.9^a^^,^ b ^.^ c	7.5±8.4^a^^,^ b ^.^ c	5.4±2.9	74.2±8.8^a^^,^ b ^.^ c	10.4±1.9	7.5±2.1^a^^,^ b ^.^ c

Note: Data are given as mean ± SD (n = 10).

a : statistically significant different from the negative control group at p<0.05;

b : statistically significant different from the parental corn group at p<0.05;

c : statistically significant different from the GM corn group at p<0.05;

d : statistically significant different from the positive control group at p<0.05.

#### 1.4. Number of splenocyte

The numbers of mice splenocytes in the positive control group were significantly lower than those in the negative control group. No other significant differences were found in the comparisons between GM corn group and the negative control group or the parental corn group (Table 8).

**Table 8:** Results of number of splenocytes, humoral immunity, cellular immunity and non-specific immunity in mice after 30 days of feeding.

Group	Negative control	Parental corn	GM corn	Positive control
Number of splenocyte (10^8^/g spleen)	7.40±1.41^d^	7.59±4.50^d^	7.15±2.51^d^	1.89±1.38^a^^,^ b ^.^ c
PFC (/10^6^ splenocytes)	30.0±18.9	29.6±25.3	31.2±32.1^d^	15.2±8.4^c^
HC_50_	256.0±64.6	257.6±25.1	265.5±32.1	227.8±48.8
IgA(µg/ml)	12.1±7.04^d^	14.32±5.16^d^	13.167.88^d^	6.09±5.88^a^^,^ b ^,^ c
IgG (µg/ml)	1808.4±959.4	1383.5±604.8	1707.9±222.8	1246.4±533.1
IgM(µg/ml)	170.4±77.4	163.8±80.7	199.0±64.1^d^	124.3±51.8^c^
IL-2(pg/ml)	3.18±1.38	2.94±0.94	2.67±0.28	2.63±0.98
IL-4(pg/ml)	2.70±0.23	2.48±0.39	2.35±0.41	2.52±0.26
IL-5(pg/ml)	17.32±7.37^b^^,^ c	12.08±4.92^a^	9.82±3.11^a^^,^ d	14.69±4.09^c^
IL-10(pg/ml)	6.57±6.03	9.93±5.38	9.71±4.96	7.88±3.27
IFN(pg/ml)	4.63±2.51	4.88±3.11	4.39±2.86	4.27±1.28
TNF(pg/ml)	9.39±4.40	7.57±1.75	7.04±1.41	8.16±1.59
ConA-induced splenocyte proliferation	0.72±0.14^d^	0.61±0.21^d^	0.58±0.23^d^	0.21±0.16^a^^,^ b ^.^ c
LPS- induced splenocyte proliferation	0.38±0.18^d^	0.38±0.20^d^	0.34±0.14^d^	0.02±0.10^a^^,^ b ^.^ c
NK cell activity	11.82±13.49	11.14±13.91	8.44±5.32	6.63±6.70
CTL cell activity	6.34±6.74	7.95±5.68	4.74±5.87	4.74±5.53
Increase in food pad thickness (mm)	5.28±3.49	4.82±3.10	4.14±2.87	5.62±4.43

Note: Data are given as mean ± SD (n = 10).

a : statistically significant different from the negative control group at p<0.05;

b : statistically significant different from the parental corn group at p<0.05;

c : statistically significant different from the GM corn group at p<0.05;

d : statistically significant different from the positive control group at p<0.05.

#### 1.5. Histological examination

**Figure 3:**
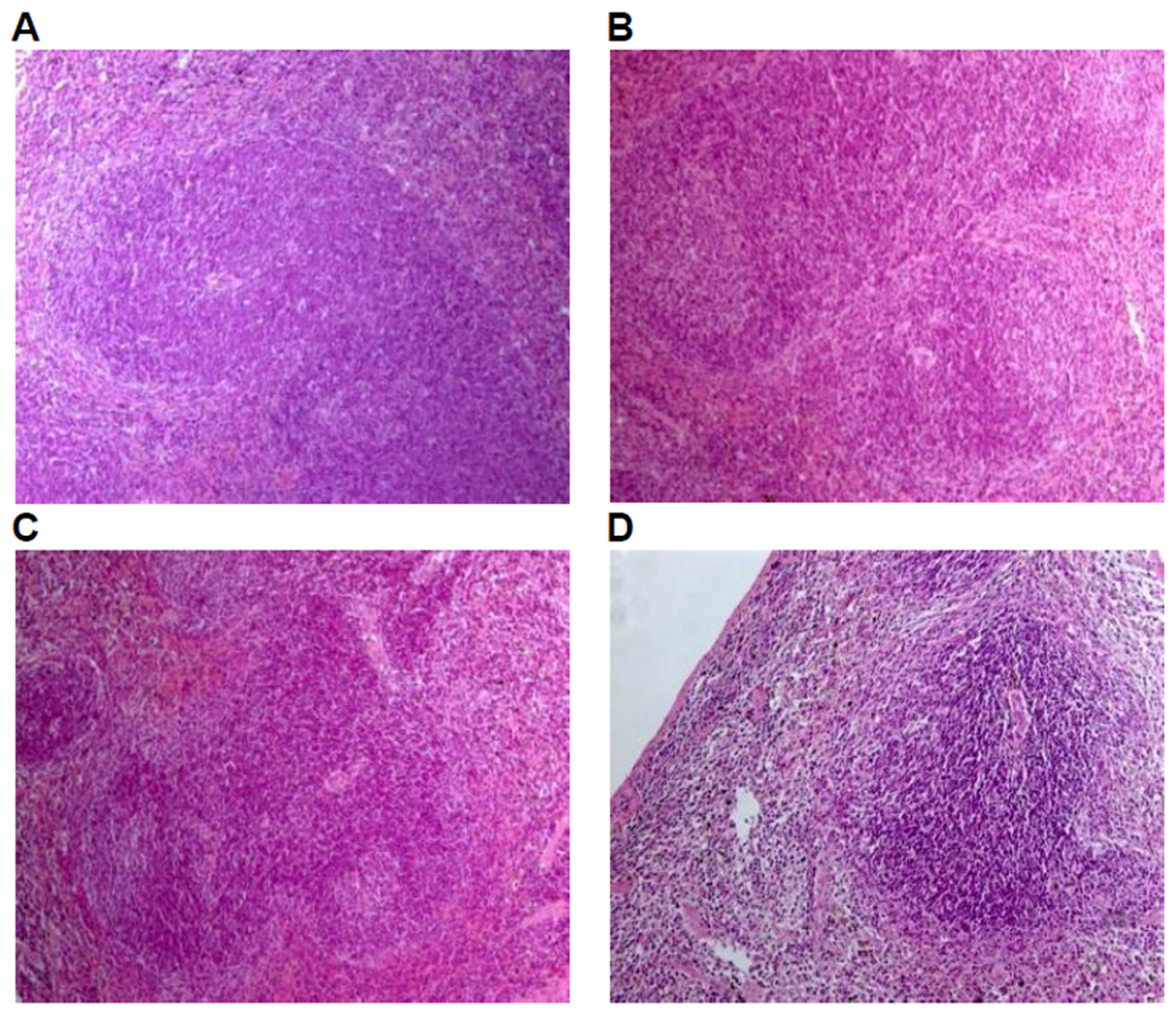
Spleen tissue of mice fed with diets containing GM corn and parental corn. Photomicrographs of histomorphological examination of spleen (100×) in female BALB/c mice in negative control group(A), non-genetically modified parental corn group(B), genetically modified corn group(C), and positive control group(D). Sections of spleen from Group A, Group B, and Group C show normal architecture. Section of spleen from Group D reveals atrophy in white pulp.

**Figure 4:**
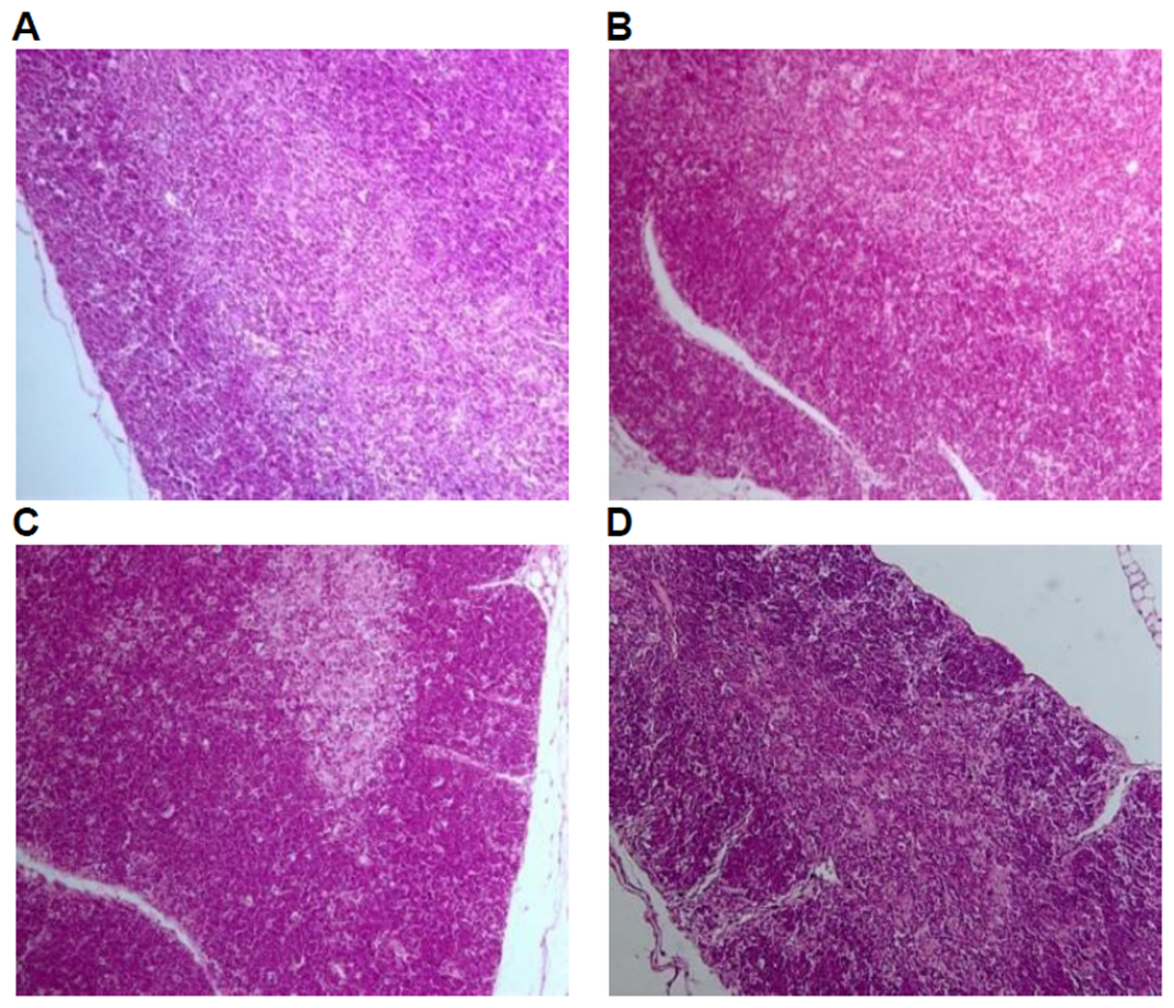
Thymus tissue of mice fed with diets containing GM corn and parental corn. Photomicrographs of histomorphological examination (100×) of thymus in female BALB/c mice in negative control group(A), non-genetically modified parental corn group(B), genetically modified corn group(C), and positive control group(D). Sections of thymus from Group A, Group B, and Group C show normal architecture. Section of thymus from Group D reveals mild atrophy.

**Figure 5:**
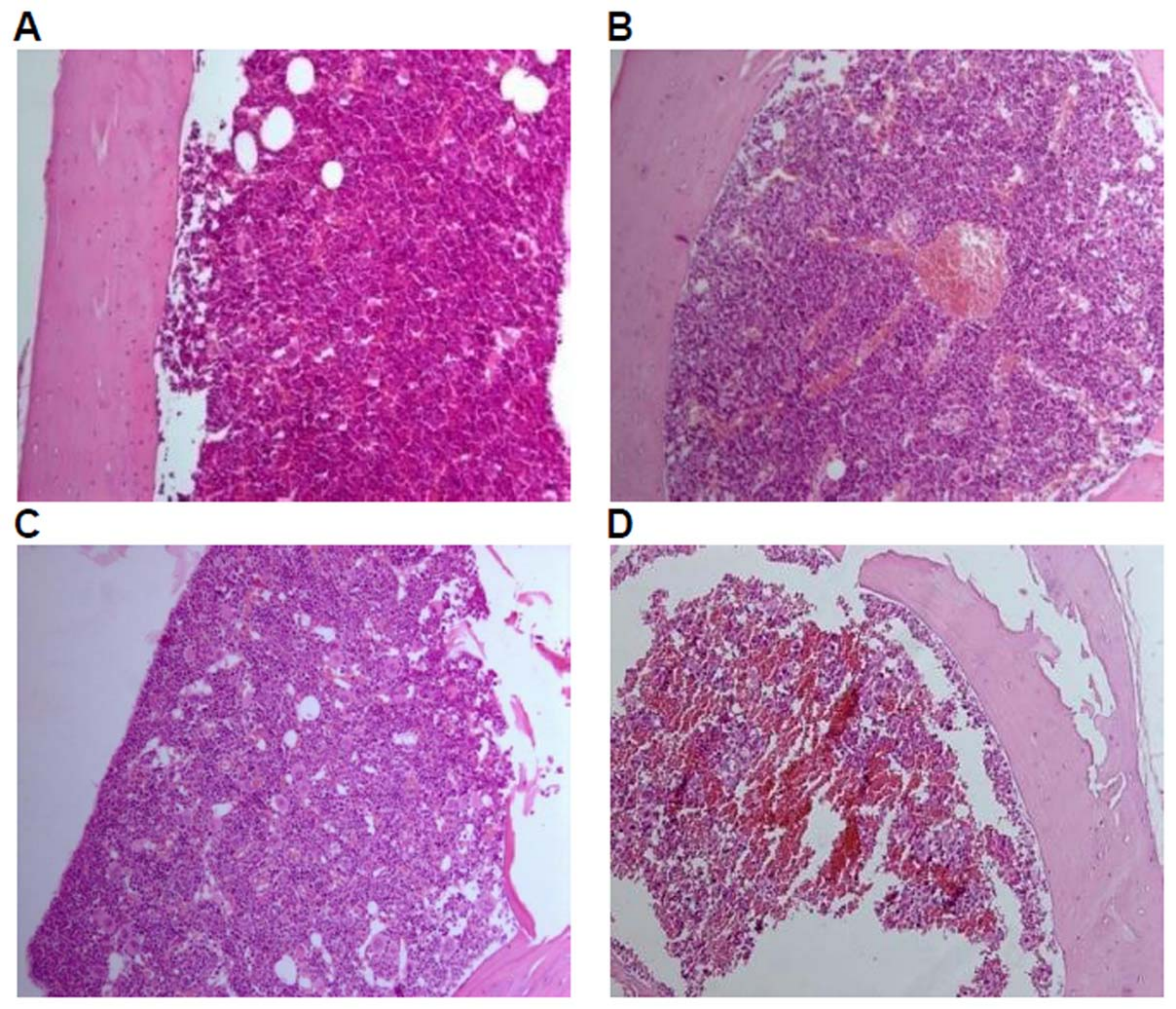
Bone marrow tissue of mice fed with diets containing GM corn and parental corn. Photomicrographs of histomorphological examination (100×) of bone marrow in female BALB/c mice in negative control group(A), non-genetically modified parental corn group(B), genetically modified corn group(C), and positive control group(D). Sections of the bone marrow from Group A, Group B, and Group C show normal architecture. Section of the bone marrow from Group D reveals decrease in the number of hematopoietic stem cells.

**Figure 6:**
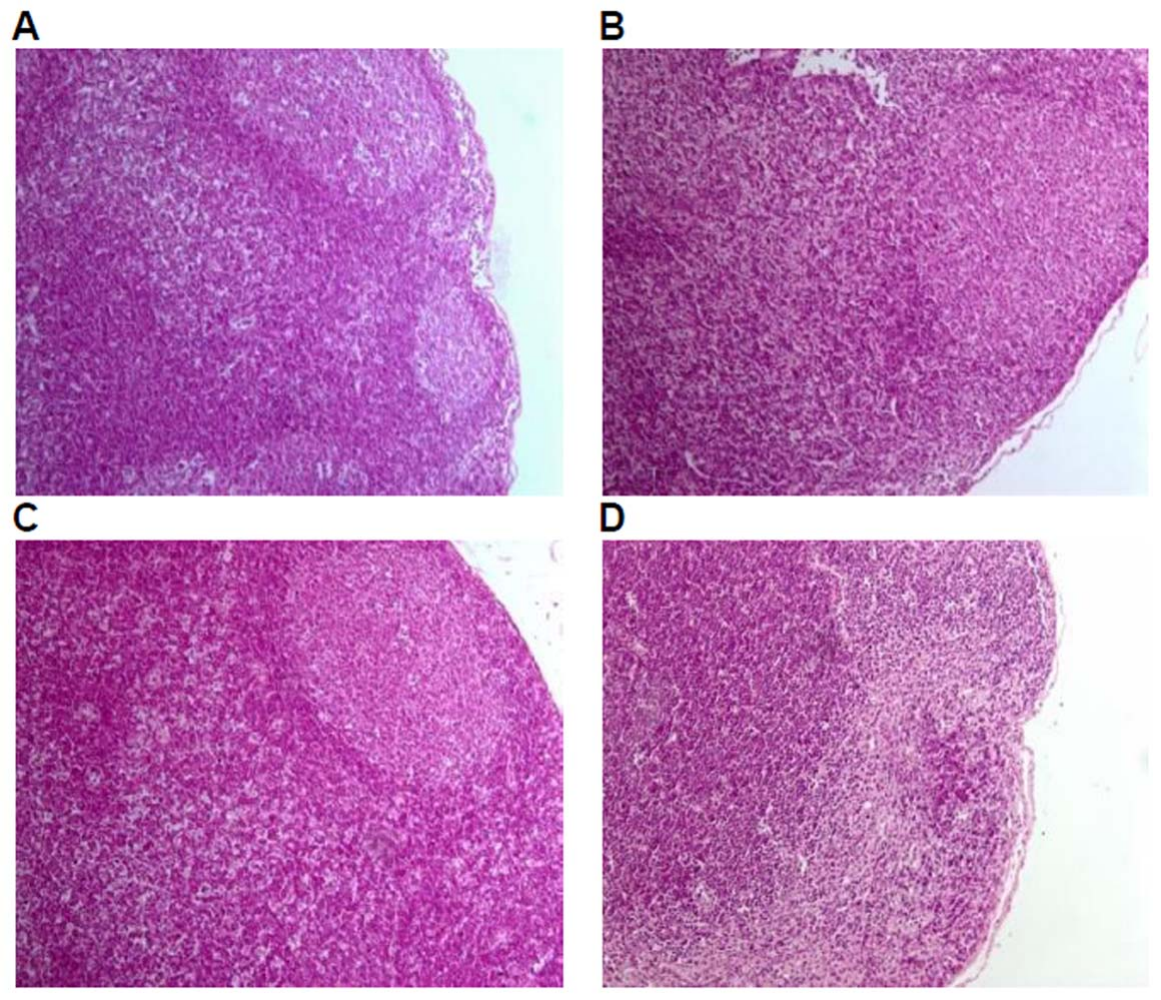
Mesenteric lymph nodes of mice fed with diets containing GM corn and parental corn. Photomicrographs of histomorphological examination (100×) of mesenteric lymph nodes in female BALB/c mice in negative control group(A), non-genetically modified parental corn group(B), genetically modified corn group(C), and positive control group(D). Sections of mesenteric lymph node from Group A, Group B, and Group C show normal architecture. Section of mesenteric lymph node from Group D reveals atrophy, unclearness of follicle structure, and fibrous tissue hyperplasia.

**Figure 7:**
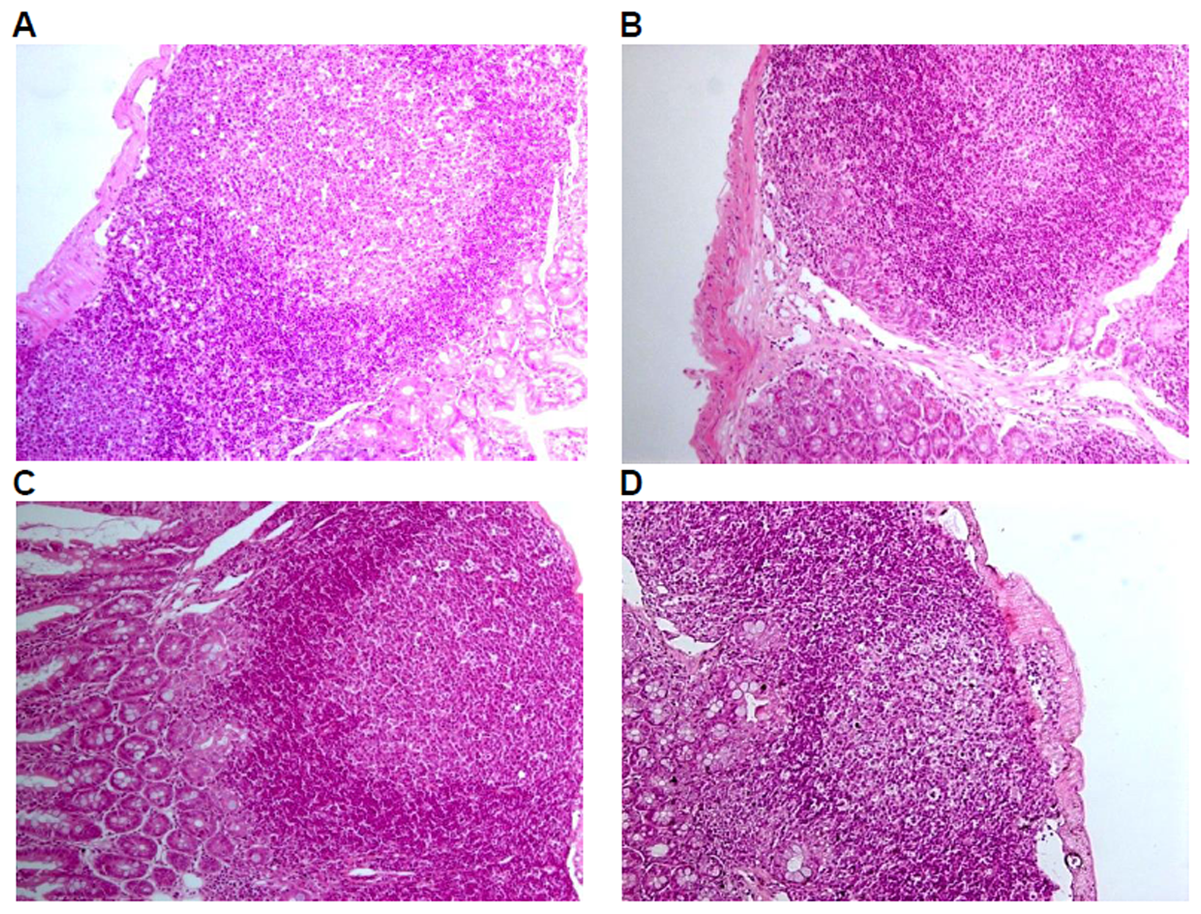
Peyer's patches of mice fed with diets containing GM corn and parental corn. Photomicrographs of histomorphological examination (100×) of Peyer's patches in female BALB/c mice in negative control group(A), non-genetically modified parental corn group(B), genetically modified corn group(C), and positive control group(D). Sections of Peyer's patch from Group A, Group B, and Group C show normal architecture. Section of Peyer's patch from Group D reveals atrophy, unclearness of follicle structure, and fibrous tissue hyperplasia.

Histological examinations were performed on all the preserved organs, pathological changes were observed only in the positive control group, including mild atrophy of thymus (10/10); decrease in the number of bone marrow (hematopoietic) stem cells (9/10); lymphopenia in white pulp of spleen, dilatation and congestion in medullary sinus of red pulp (10/10), of which 7 cases of mild white pulp atrophy, 3 cases of moderate white pulp atrophy; lymph node atrophy(cervical lymph nodes, axillary lymph nodes, mesenteric lymph nodes, and Peyer's patches), unclearness or disappearance of follicle structure, fibrous tissue hyperplasia (9/10). No pathological changes of liver, kidney, and mucous membrane of small intestine in the positive control group and of liver, kidney,spleen, thymus, mucous membrane of small intestine and lymph glands including cervical lymph nodes, axillary lymph nodes, mesenteric lymph nodes, and Peyer's patches were observed in the GM corn group, the negative control group, and the parental group (Figure 3, 4, 5, 6, 7).

### 2. Humoral immunity

#### 2.1. PFC and HC_50_

The PFC assay and hemolysis test were used to detect humoral immunity toxicity. A single dose of CY (200 mg/kg bw) induced reduction in PFC/10^6^ splenocytes and HC_50_ when compared with the negative control group, but not statistically significant. No significant difference in the GM corn group was observed when compared with those in the negative control group and the parental corn group (Table 8).

#### 2.2. Serum immunoglobulins

The serum IgA levels in the positive control group were significantly lower than those in the negative control group. And no significant differences were found with respect to serum levels of IgA, IgM, and IgG between GM corn group and the negative control group or the parental corn group (Table 8).

#### 2.3. Serum cytokines

No significant differences of serum IL-2, IL-4, IL-5, IL-10, IFN-γ and TNF levels of mice in the positive control group were observed when compared with those in the negative control group. Serum IL-5 levels in the GM corn group and the parental corn group were significantly lower than those in the negative control group, but such difference was not found between the GM corn group and the parental corn group (Table 8).

### 3. Cellular immunity-Splenocyte proliferation, CTL cell activity, and DTH reaction

The mitogen-induced splenocyte proliferation, CTL cell activity, and DTH reaction were used to detect cellular immunity. At a single dose of 200 mg/kg, CY elicited a suppressive effect in ConA-induced T lymphocyte proliferation, LPS-induced B lymphocyte proliferation, and the reaction of DTH when compared with the positive control group. There was no significant change of the activity of CTL cells between the positive control group and the negative control group. No significant differences of these cellular immunity parameters in the GM corn group were observed when compared with those in the negative control group and the parental corn group (Table 8).

### 4. Non-specific immunity-NK cell activity and phagocytic index

NK cell activity test and mice carbon-clearance test were used to detect non-specific immunity. No significant change of the activity of NK cells and phagocytic index of mice was found among all groups (Table 8).

## Discussion

The immune system is very sensitive to a variety of chemical and physical stressors and as such it can be used as a tool to examine the subclinical effects of chemical exposure [20]. The purpose of this study was to evaluate the immunomodulatory effects of the GM corn modified with *Bt Cry1Ah gene* in BALB/c mice for 30 days. Immunopathology parameters were examined, and assays reflecting the function of humoral immunity, cellular immunity and non-specific immunity were conducted.

Cyclophosphamide can reduce the number of circulating lymphocytes and impair the function of humoral and cellular immune response, and was commonly used as immunosuppressive agent in immunotoxicology studies. In this study, a single dose of CY (200 mg/kg bw) was administered via intraperitoneal injection as a positive control. Some immune changes were found including significant decreases in spleen, thymus and lymph gland weights, statistically significant decreases in leucocyte absolute value and partial differential leucocyte value and percentage, serum IgA, the percentages of T cells of peripheral blood lymphocytes, the number of splenocyte, LPS- and ConA-induced splenocyte proliferation, DTH. Significant increases of the percentages of B/Th cells, Th/Ts ratio of peripheral blood lymphocytes, serum ALT level, NEUT percentage of WBC, MPV level were detected, some histopathology changes in thymus, spleen, bone marrow and lymph nodes were also observed. The results of this study showed that the administration of cyclophosphamide led to significant inhibition of most of the immunopathology and functional parameters. So the animal model used to evaluate the immunetoxic effect was successful. As reported by Luster et al., cyclophosphamide provided an indicator of assay of effects on multiple cell types (e.g., B cells, T cells, macrophages), and of relative immunotoxic potency for the chemical being tested [20]. A guideline on immunotoxicity of US EPA (OPPTS870.7800) described that a known immunosuppressant (e.g., cyclophosphamide) is useful in the interpretation of the results or verification of the assay sensitivity, and should be included in the study [21]. Many reported studies together with our findings demonstrate that cyclophosphamide has the potential to induce immunosupression [22]–[24].

In spite of the extensive research in the field of GM crops, there is a still increasing requirement from consumers and regulative authorities for guideline tests to assess the safety of genetically modified foods [25]. In recent years, reviews in the literature have addressed the need for guidelines for safety testing of GMOs at all levels from construction and transfer of gene products to the final toxicity testing in animal model [26]. However, up to now, assessment of the immunotoxicological effects of GMOs has mainly focused on the allergenic potential of genetically modified proteins, whereas general immunotoxicological investigations of whole GMOs are rarely described in the literature [6].

Results of our present study indicate that there were no significant differences on spleen and thymus weights, WBC count, immune function parameters, histological examination of immune organ of BALB/c mice in the GM corn group when compared with mice in the parental corn group, and the negative control group. Body weight on week 4, MON value, serum IL-5 level in blood of mice in GM corn group were lower than those in the negative control group. However, the similar changes were also observed in the parental corn group. So these changes observed were not attributed to GM corn consumption. The possible reason for the decrease of body weight in the negative control group might be the intraperitoneal injection of saline. In terms of hematology parameters, though MON value in the GM corn group decreased, its percentage had no significant change. Serum cytokine change could be indicative of some kind of humoral immunity damage, however, no significant differences on humoral immunity function were observed through PFC assay and hemolysis test. Similar to our findings, Kroghsbo et al. reported that no adverse immunotoxicological effects were found in rats administered by the transgenic Bt rice expressing Cry1Ab protein after 28 or 90 days [6]. However, the differences in immune response have previously been observed in fish following Bt maize consumption [27], and in mice after 30 and 90 days of Bt maize consumption [7]. The inconsistency in results between studies is likely to be due to the use of different animal models and the different levels of Bt protein in the diets. In our study, Cry1Ah protein was expressed in GM corn at low level (<0.05% of total protein, 1.92∼3.08 µg per gram of corn) confirmed by ELISA kits (EnviroLogix,USA).

In this study, the levels of main nutrients and active ingredients in the GM corn and the parental corn were detected by Institute of Plant Protection, Chinese Academy of Agricultural Sciences. No statistically significant differences were found in the nutrients (including protein and amino acids, fat and fatty acid, carbohydrate, fiber, minerals, vitamins, etc.), anti-nutrients (raffinose, phylic acid, trypsin inhibitor), moisture, ash content, and other natural ingredients between the GM corn and the parental corn.

In our previous allergenicity studies, we analysed the sequence homoloyg of amino acids of Cry1Ah protein with known potent allergens by the bioinformatics tools. The bioinformatics results show that Cry1Ah protein had no homology. Furthermore, we analyzed the digestive stability of Cry1Ah protein in the simulated gastric fluid and intestinal fluid in vitro by the STD-PAGE analysis. The results show that the bands of Cry1Ah protein in simulated gastric fluid gradually became lighter after periods of 0 sec, 15 sec, 2 min. The band of cry1Ah completely disappeared after 10 minutes. In the simulated intestinal fluid, the band of Cry1Ah protein was still visible up to 60 min. Thus, Cry1Ah protein was able to be digested in simulated gastric fluid, but was very difficult to be digested in simulated intestinal fluid. In our earlier allergenicity study of Cry1Ah protein in Brown Norway rats, the results showed that Cry1Ah protein was unable to induce significant increase of serum IgE and histamine level. According to the FAO/WHO decision tree, when negative results are obtained in both pepsin digestibility assay and animal model experiments, the expressed protein is unlikely to become an allergen [28].

In conclusion, the corn genetically modified with *Bt Cry1Ah* gene is considered consistent with the parental corn in terms of immunopathology, humoral immunity, cellular immunity and non-specific immunity. No adverse immunotoxicological effects of GM corn with *Bt Cry1Ah* gene were found when feeding mice for 30 days.
